# Stability Analysis of Navier–Stokes–Voigt Fluids in Porous Media with Slippery Effect

**DOI:** 10.3390/nano16060367

**Published:** 2026-03-17

**Authors:** Jing Shi, Jiayu Zhang, Quansheng Liu, Zhaodong Ding, Ruigang Zhang

**Affiliations:** 1 School of Mathematical Sciences, Inner Mongolia University, Hohhot 010030, China; 2 Inner Mongolia Key Laboratory of Mathematical Modeling and Scientific Computing, Hohhot 010030, China

**Keywords:** Navier–Stokes–Voigt fluids, slip, linear stability, Chebyshev spectral collocation

## Abstract

This paper investigates the linear stability of Navier–Stokes–Voigt (NSV) fluid flow in a channel filled with a homogeneous porous medium under general asymmetric slip boundary conditions. This study bridges the research gap between idealized theoretical models (uniform coating) and realistic engineering surfaces in superhydrophobic channels. In practice, manufacturing defects often lead to non-uniform slip distributions. By solving the generalized eigenvalue problem using the Chebyshev spectral collocation method, we quantify the sensitivity of the critical Reynolds number to symmetry breaking. The results reveal that symmetric slip achieves optimal stability, whereas symmetry breaking causes a significant destabilizing effect. Energy analysis clarifies the physical origin of this instability. Furthermore, we find that increasing the porous medium permeability parameter or the Voigt regularization parameter effectively counteracts the slip-induced instability. Specifically, flow stability can be restored even under highly asymmetric slip conditions if the porous damping or the viscoelastic regularization effect is sufficiently strong. This implies that inevitable manufacturing defects in engineering can be compensated for by optimizing the porous medium matrix.

## 1. Introduction

For decades, fluid system stability research has remained a highly compelling and prominent direction in fluid mechanics. Specifically, the stability of incompressible channel flow is a fundamental problem in fluid mechanics, which has been extensively studied from a theoretical perspective [1]. Beyond its theoretical significance, this problem has gained increasing attention in various engineering applications, such as microfluidic transport, filtration processes in porous media, and lubrication systems [2]. The roots of this research trace back to Reynolds’ pioneering experiment in 1883 [3]. This work marked the start of modern fluid stability studies and inspired Orr’s [4] and Sommerfeld’s [5] theoretical investigations. Working independently, they considered small-amplitude traveling-wave perturbations imposed on steady parallel flows. From this, they derived the classic Orr–Sommerfeld equation. Subsequent scholars conducted in-depth studies on various flow configurations. These include planar Poiseuille flow (PPF), planar Couette flow (PCF), Hagen–Poiseuille flow (HPF), and boundary layer flow [6]. These efforts have progressively refined the theoretical framework of fluid stability.

However, traditional fluid stability studies often rely on the no-slip boundary condition assumption. Research on small-scale fluid flows shows that the commonly assumed “smooth surface” fails to meet hydrodynamic smoothness criteria. This leads to significant deviations when the classical no-slip model describes micro- and nano-scale flows. As early as 1823 [7], Navier first proposed a mathematical model for slip flow:

$$u_{s}={\left.\lambda \frac{\partial u}{\partial y}\right|}_{y=0}$$

In this equation, λ denotes the slip length, $u_{s}$ is the slip velocity component in the flow direction, y=0 indicates the wall position, and *y* represents the wall normal direction. This condition establishes that the tangential velocity near the surface is proportional to the surface shear rate. When λ=0, it corresponds to the no-slip condition (i.e., u=0). When λ→∞, it represents an ideal slip surface. Subsequently, after a century of theoretical exploration, Vinogradova [8] provided a comprehensive review and further quantified the slip phenomena in uniform high-hydrophobicity systems through experimental comparison. Experimental observations showed a slip length of λ≈30±20nm for surfaces with contact angles >90. With advances in materials science, research on nanoscale superhydrophobic surfaces has deepened. Tretheway and Meinhart [9] used micrometer-resolution particle image velocimetry (μ-PIV) to measure significant apparent slip in hydrophobic microchannels coated with a single layer of OTS. The measured slip length reached λ≈1μm. Choi’s [10] precise experiments demonstrated that slip length on hydrophobic surfaces increases approximately linearly with shear rate.

Additionally, under identical pressure differentials, flow rates in hydrophobic channels consistently exceed those in hydrophilic channels. This provides direct evidence of the positive correlation between slip length and drag reduction effects. These properties have proven valuable in practical applications, such as efficient transport in microfluidic chips and sweat dissipation in wearable electronics [11]. Regarding flow stability under slip boundary conditions, academic debate once existed. Chu [12] incorrectly concluded that critical Reynolds number decreases with increasing slip length. This error stemmed from improper boundary condition treatment. In contrast, Lauga and Cossu [13] and Spille [14] obtained opposite results. Later, Webber [15] used the accurate Chebyshev tau numerical method to verify the stabilizing effect of slip boundaries. This finding aligned with Lauga et al.’s conclusions.

Notably, most previous channel flow stability studies focused on Newtonian fluids. Non-Newtonian fluids—such as polymer solutions, blood, and emulsions—are now widely used in engineering. As a result, the Newtonian fluid assumption can no longer meet practical demands. Research on non-Newtonian slip flow has thus emerged as a hot topic. For example, Patlazhan and Vagner [16] investigated shear-thinning fluid flow on superhydrophobic surfaces. Their work provided a theoretical basis for designing high-efficiency drag-reducing microfluidic devices. Ferrás et al. [17] systematically analyzed viscoelastic fluid behavior under slip boundary conditions. Their findings have been applied in polymer processing, lubrication, and coating technologies.

The classical Navier–Stokes equations serve as the core theoretical framework for describing fluid motion, but their three-dimensional well-posedness remains an unsolved challenge. Potential singularities severely restrict theoretical analysis and numerical computation, prompting mathematical regularization as a critical research direction and leading to the development of the Navier–Stokes–Voigt (NSV) model. Notably, the NSV model is a simplified version of the second-grade fluid model [18,19], retaining key viscous and elastic stress terms related to strain rate and its time derivative while omitting higher-order nonlinear terms. This simplification ensures mathematical tractability while capturing core dissipative and regularization effects, making it suitable for stability analysis under complex boundary conditions. In his pioneering work, Oskolkov first proposed the NSV equations to describe the motion of viscoelastic incompressible fluids following the Kelvin–Voigt model. He also established a comprehensive framework covering several important viscoelastic fluid models, including Oldroyd fluids and Maxwell fluids. Unlike classical models such as Oldroyd-B (derived from polymer dynamics), the Navier–Stokes–Voigt (NSV) model introduces an elastic stress term proportional to the time derivative of the strain rate into its constitutive equations. This creates a highly tractable mathematical framework. While this simplification prevents the model from predicting typical viscoelastic phenomena (e.g., shear thinning and normal stress differences), it effectively suppresses singularities inherent in the original Navier–Stokes equations. Subsequently, Oskolkov and Shadiev [20] rigorously proved the existence of global classical solutions for initial-boundary value problems of Oldroyd and Kelvin–Voigt fluids over infinite time intervals. They systematically summarized the motion equations for these viscoelastic fluids, laying the theoretical foundation for NSV model applications. Although Oskolkov’s work initially confirmed the NSV model’s mathematical regularization properties, its connection to the original Navier–Stokes equations required explicit verification. In 2012, Berselli and Bisconti [21] conducted the first systematic analysis of the limit behavior of Euler–Voigt and Navier–Stokes–Voigt (NSV) models. Through rigorous proof, they showed that under appropriate initial conditions, as the Voigt parameter approaches zero, the model solutions strongly converge to those of the original Euler or Navier–Stokes equations. This conclusion provides core theoretical support for applying Voigt-type models in turbulence simulation and computational fluid dynamics. It also transformed these models from purely mathematical tools to practical research instruments for flow problems.

Building on Oskolkov’s foundational work and subsequent theoretical advancements, the NSV model has gradually been applied to stability analysis in complex scenarios—such as flow through porous media. Here, the influence of slip boundary conditions has become a key research focus. Straughan et al. [22] conducted pioneering studies on the effect of slip boundary conditions on Poiseuille flow instability in Brinkman-type porous media. Based on the Brinkman porous medium model, they derived flow control equations incorporating slip boundary conditions. They introduced two key parameters: a dimensionless slip coefficient (characterizing slip length) and a Darcy friction coefficient (characterizing porous medium resistance). To ensure the accuracy of critical parameter calculations, they validated their approach using two Chebyshev configuration methods, the finite element method, and the QZ algorithm for solving eigenvalue problems. Their study yielded several important findings: With fixed porous medium parameters, increasing the slip coefficient significantly raises the critical Reynolds number. This indicates that slip boundaries exert a strong stabilizing effect on flow, delaying laminar–turbulent transition. With fixed slip coefficient, increasing the porous medium resistance parameter (reducing porosity and increasing resistance) further increases the critical Reynolds number. This synergizes with the stabilizing effect of slip boundaries. As slip length increases, the critical wave number decreases, indicating sparser periodic structures in flow disturbances. As the porous medium coefficient increases, the critical wave number first decreases and then increases, with an inflection point in the 2–3 range. This research clarified the stabilizing effects of increased Darcy friction coefficient and slip length on flow stability. The Brinkman model is suitable for describing flow in large-pore porous media, while the Darcy model applies to small-pore scenarios. Together, they provide a foundation for boundary condition analysis in Navier–Stokes–Voigt fluid stability studies within porous media.

While the stability of Navier–Stokes–Voigt (NSV) fluids has been studied in various contexts, most research focuses on clear fluid channels. Recently, Shankar and Shivakumara [23] extended this analysis to porous media; nevertheless, their investigation was limited to idealized symmetric slip conditions ($\lambda_{1}=\lambda_{2}$). In reality, this assumption of perfect symmetry often fails to capture engineering complexities for two key reasons. First, unavoidable manufacturing tolerances in superhydrophobic coatings—such as uneven spray thickness—inevitably result in non-uniform slip lengths. This fabrication-induced asymmetry is a phenomenon that symmetric models cannot accurately describe. Second, in advanced microfluidic design, intentionally using asymmetric coatings (e.g., $\lambda_{1}\neq \lambda_{2}$) allows for the “directional regulation” of flow stability. This approach offers significantly greater flexibility than symmetric configurations. Consequently, the impact of slip asymmetry on flow stability remains a critical but unresolved issue.

To bridge the gap between idealized theory and these practical engineering scenarios, this study performs a comprehensive linear stability analysis of NSV fluid flow under general asymmetric slip conditions. The novel contributions of this work are threefold. First, instead of assuming perfect symmetry, we quantify the sensitivity of the critical Reynolds number to slip asymmetry using the Chebyshev spectral collocation method. This establishes a theoretical basis for evaluating the allowable range of manufacturing defects in engineering design. Second, we investigate the coupling mechanism between the fluid’s viscoelastic regularization (Voigt term) and the Darcy drag of the porous medium in the presence of asymmetry. Finally, we propose a strategy to counteract the instability caused by manufacturing-induced asymmetry. This is achieved by optimizing the porous medium permeability parameter (*M*) or the Voigt parameter (Λ).

The remainder of this paper is organized as follows: Section 2 establishes the mathematical framework for NSV fluid flow in porous media. Specifically, Section 2.1 and Section 2.2 present the governing equations and the steady base flow solutions under varying slip conditions; Section 2.3 derives the modified Orr–Sommerfeld perturbation equation; Section 2.4 discusses the analogy between asymmetric slip flow and Poiseuille–Couette flow; and Section 2.5 formulates the disturbance energy budget equation. Finally, Section 2.6 justifies the selection of physical parameters based on experimental references. Section 3 discusses the numerical results and physical mechanisms. It begins with the solution of the generalized eigenvalue problem via the Chebyshev spectral collocation method and code validation. Subsequently, the neutral stability curves and critical Reynolds numbers are analyzed with respect to key parameters, including slip asymmetry, porous medium permeability, and the Voigt parameter. Finally, based on the global energy budget analysis, the physical origins of slip-induced instability and the corresponding compensation mechanisms are thoroughly investigated. Section 4 summarizes the conclusions.

## 2. Mathematical Formulation

**Figure 1:**
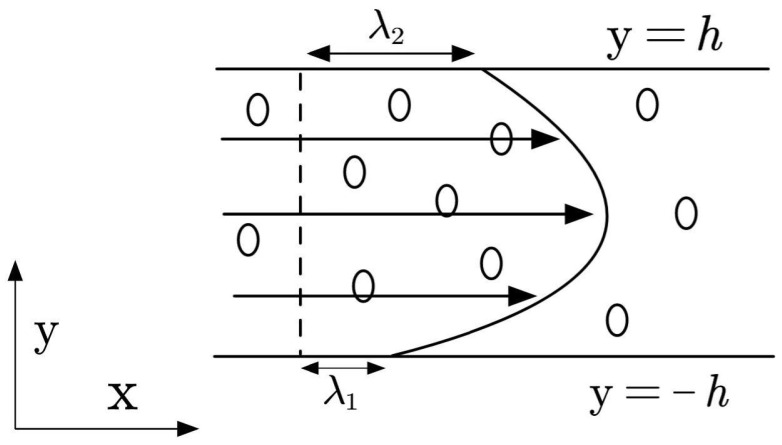
Model of NSV fluid in a porous media channel.

Consider a layer of incompressible Navier–Stoke–Voigt fluid confined between two infinitely long rigid parallel plates. The distance between the plates is 2h, and the flow is driven by a constant pressure gradient, with the system embedded in a uniform porous medium. According to Squire’s theorem, for the basic flow of incompressible laminar flow, if a two-dimensional unstable disturbance exists, there must be a three-dimensional unstable disturbance with a lower critical Reynolds number. Consequently, our stability analysis only needs to consider the two-dimensional model. A Cartesian coordinate system is adopted, where the *x*-axis is parallel to the flow direction, the *y*-axis points upward, and the origin is located at the center of the channel, as illustrated in Figure 1.

Consider a layer of incompressible Navier–Stokes–Voigt fluid confined between two infinitely long rigid parallel plates. The distance between the plates is 2h, and the flow is driven by a constant pressure gradient, with the system embedded in a uniform porous medium. It is well known that Squire’s theorem does not universally apply to all non-Newtonian fluids. However, because the viscoelastic Voigt regularization term is mathematically linear, recent rigorous derivations by Shankar and Shivakumara [23] have established that the classical Squire’s transformations remain perfectly valid for the NSV fluid model. According to this validated Squire’s theorem, if a three-dimensional unstable disturbance exists, there must be a two-dimensional unstable disturbance with a lower critical Reynolds number. Consequently, our stability analysis rigorously only needs to consider the two-dimensional model. A Cartesian coordinate system is adopted, where the *x*-axis is parallel to the flow direction, the *y*-axis points upward, and the origin is located at the center of the channel, as illustrated in Figure 1.

Herein, we generalize the model for the flow of viscoelastic fluids in channels: the fluid exhibits a specific slip length $\lambda_{2}$ at the upper plate and a specific slip length $\lambda_{1}$ at the lower plate. When $\lambda_{2}=\lambda_{1}=0$, the flow corresponds to Poiseuille flow; when $\lambda_{2}=\lambda_{1}\neq 0$, it represents symmetric flow with slip conditions; and the configurations $\lambda_{1}>\lambda_{2}$ and $\lambda_{1}<\lambda_{2}$ are physically equivalent. To avoid redundancy and maintain mathematical generality, we only discuss the general asymmetric case where $\lambda_{1}\neq \lambda_{2}$.

The mathematical formulation of the above model is given by the continuity equation and the Navier–Stokes equations:

(1) ∇·V=0.

(2) $$\rho \left[\frac{\partial V}{\partial t}+\left(V\cdot \nabla \right)V\right]=\nabla \cdot \tilde{T}-\frac{\mu }{k}V_{d}.$$

$\tilde{T}$ is the stress tensor of the zeroth-order Navier–Stokes–Voigt fluid as given by Oskolkov [24] and Straughan [25].

(3) $$\tilde{T}=-p\tilde{I}+\rho \tilde{\lambda }\frac{\partial }{\partial t}\left(\nabla V+\nabla V^{tr}\right)+\mu \left(\nabla V+\nabla V^{tr}\right).$$

Here, $\tilde{I}$ is the unit tensor and tr denotes the transpose of the indicated tensor. In addition, we re-scale the elastic parameter and we replace $2\tilde{\lambda }$ simply with $\tilde{\lambda }$. From the Dupuit–Forchheimer relation $V_{d}=\phi V$ [26], we get

(4) $$\rho \left(1-\tilde{\lambda }\nabla^{2}\right)\frac{\partial V}{\partial t}+\rho \left(V\cdot \nabla \right)V=-\nabla P+\mu \nabla^{2}V-\frac{\mu \phi }{k}V.$$

Herein, (V=(u,v)) denotes the velocity vector, ρ represents the fluid density, $V_{d}$ is the Darcy velocity, *t* stands for time, and p denotes the pressure. The permeability *k* characterizes the conductivity of the porous medium: a larger *k* corresponds to a smaller resistance to fluid flow, while a smaller *k* leads to a larger flow resistance. The porosity ϕ of the porous medium refers to the ratio of the pore volume to the total volume of the porous medium; a smaller ϕ results in narrower effective flow channels. $\tilde{\lambda }$ is defined as the Kelvin–Voigt parameter. Under all considered cases, the half-width h of the channel is selected as the length scale, and the maximum fluid velocity $U_{0}$ is adopted as the velocity scale to normalize $\tilde{\lambda }$ and *k*. The appropriate dimensionless treatment is given as follows:

(5) $$x^{*}=\frac{x}{h},t^{*}=\frac{tU_{0}}{h},V^{*}=\frac{V}{U_{0}},p^{*}=\frac{p}{\rho U_{0}^{2}}.$$

Substituting Equation (5) back into Equation (4) and omitting the asterisks, the component forms of the nondimensionalized Navier–Stokes equations and continuity equation are obtained as:

(6) ∂u∂x+∂v∂y=0,1−Λ∇2∂u∂t+u∂u∂x+v∂u∂y=−∂P∂x+1Re∇2u−M2Reu,1−Λ∇2∂v∂t+u∂v∂x+v∂v∂y=−∂P∂y+1Re∇2v−M2Rev.

Herein, μ denotes the dynamic viscosity coefficient of the fluid. Re represents the Reynolds number. It roughly describes the ratio of inertial forces to viscous forces in the flow. Its specific expression is given by Re=ρU0hμ. As a fundamental indicator, this parameter is crucial for determining the flow regime and evaluating flow stability. *M* is defined as the permeability parameter. It is specifically used to quantify the fluid’s ability to penetrate the porous medium. The magnitude of M is positively correlated with permeability: a larger M indicates stronger resistance for the fluid to penetrate the pores of the porous medium, while a smaller M means weaker permeation resistance for the fluid. Mathematically, it is expressed as $M=h\sqrt{\frac{\phi }{k}}$, where *h* is the channel half-height, ϕ is the porosity of the porous medium, and *k* is the permeability of the porous medium. This parameter directly reflects the inhibitory or facilitatory effect of the porous medium’s structure on fluid flow. Λ refers to the Kelvin–Voigt parameter. Its intrinsic physical meaning is to describe the ratio of elastic forces to viscous forces during the flow of the Navier–Stokes–Voigt (NSV) fluid. As a key dimensionless number characterizing the viscoelastic properties of the fluid, its mathematical expression is $\Lambda =\frac{\tilde{\lambda }}{h^{2}}$, where $\tilde{\lambda }$ is the dimensional Kelvin–Voigt elastic parameter and *h* is the channel half-height. The magnitude of Λ directly affects the fluid’s response to perturbations. Generally, a larger Λ enhances the fluid’s ability to suppress unstable flow modes, thereby exerting a significant regulatory effect on flow stability.

### 2.1. Base Flow Analysis

To establish the stability equations under asymmetric slip boundaries, determining the base flow velocity profile for the NSV fluid is essential. The detailed derivation proceeds as follows. Since the Navier–Stokes–Voigt terms in Equation (6) do not contribute to the base flow solution, and the base flow is a fully developed, unidirectional and steady laminar flow, its velocity vector can be expressed as $V=\left(u_{b}\left(y\right),0\right)$. Here, the subscript “b” denotes the basic state. Substituting this velocity vector into Equation (6) yields a second-order ordinary differential equation.

(7) d2ubdy2−M2ub=RedPdx.

Let ∂P∂x=−coshMcoshM−1M2Re represent the pressure of the undisturbed fluid motion. To solve for the base flow, Equation (7) is solved simultaneously with the boundary conditions:

(8) $$u_{b}-\lambda_{1}\frac{du_{b}}{dy}=0\;\;\mathrm{on}\;\;y=-1.$$

(9) $$u_{b}+\lambda_{2}\frac{du_{b}}{dy}=0\;\;\mathrm{on}\;\;y=1.$$

The resulting solution for the base flow is as follows:

(10) uby=AcoshMy+BsinhMy−Re∂P∂xM2.

where

A=Re∂P∂xM22sinhM+λ1+λ2McoshM1+λ1λ2M2sinh2M+λ1+λ2Mcosh2M.

B=Re∂P∂xM2λ1−λ2MsinhM1+λ1λ2M2sinh2M+λ1+λ2Mcosh2M.

### 2.2. Boundary Condition

#### 2.2.1. Asymmetric Slip Conditions

When $\lambda_{1}\neq \lambda_{2}\neq 0$

(11) $$\begin{array}{cc} u_{b}\left(y\right) & =\frac{\cosh(M)}{\cosh(M)-1}\left[-\frac{\left(\lambda_{1}-\lambda_{2}\right)M\sinh\left(M\right)}{\left(1+\lambda_{1}\lambda_{2}M^{2}\right)\sinh\left(2M\right)+\left(\lambda_{1}+\lambda_{2}\right)M\cosh\left(2M\right)}\sinh\left(My\right)\right.\\ \; & \;\left.-\frac{\left[2\sinh\left(M\right)+\left(\lambda_{1}+\lambda_{2}\right)M\cosh\left(M\right)\right]}{\left(1+\lambda_{1}\lambda_{2}M^{2}\right)\sinh\left(2M\right)+\left(\lambda_{1}+\lambda_{2}\right)M\cosh\left(2M\right)}\cosh\left(My\right)+1\right]. \end{array}$$

When M→0

(12) $$u_{b}=\frac{-\left[2+\left(\lambda_{1}+\lambda_{2}\right)\right]y^{2}-2\left(\lambda_{1}-\lambda_{2}\right)y+2+3\left(\lambda_{1}+\lambda_{2}\right)+4\lambda_{1}\lambda_{2}}{2+\lambda_{1}+\lambda_{2}}.$$

When $\lambda_{2}=0$ and $\lambda_{1}=\lambda_{0}\neq 0$

(13) $$\begin{array}{cc} u_{b}\left(y\right) & =\frac{\cosh(M)}{\cosh(M)-1}\left(-\frac{2\sinh\left(M\right)+\lambda_{0}M\cosh\left(M\right)}{\sinh\left(2M\right)+\lambda_{0}M\cosh\left(2M\right)}\cosh\left(My\right)\right.\\ \; & \;\left.-\frac{\lambda_{0}M\cosh\left(2M\right)}{\sinh\left(2M\right)+\lambda_{0}M\cosh\left(2M\right)}\sinh\left(My\right)+1\right) \end{array}$$

When M→0

(14) $$u_{b}\left(y\right)=1+\frac{2\lambda_{0}\left(1-y\right)}{2+\lambda_{0}}-y^{2}.$$

#### 2.2.2. Symmetric Slip Conditions

If $\lambda_{1}=\lambda_{2}=\lambda_{0}$, then $u_{b}\left(y\right)$ takes the form:

(15) $$u_{b}\left(y\right)=\frac{\cosh\left(M\right)}{\cosh\left(M\right)-1}\left(-\frac{\cosh\left(My\right)}{\cosh\left(M\right)+\lambda_{0}M\sinh\left(M\right)}+1\right).$$

When M→0

(16) $$u_{b}=1+2\lambda_{0}-y^{2}$$

However, if $\lambda_{1}=\lambda_{2}=0$, $u_{b}\left(y\right)$ simplifies to:

(17) $$u_{b}\left(y\right)=\frac{\cosh\left(M\right)-\cosh\left(My\right)}{\cosh\left(M\right)-1}.$$

When $M=\lambda_{1}=\lambda_{2}=0$

(18) $$u_{b}\left(y\right)=1-y^{2}.$$

**Figure 2:**
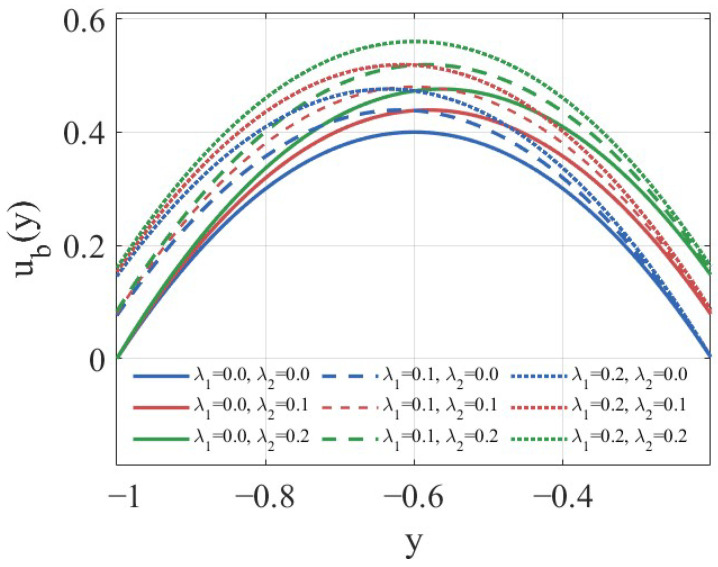
The base flow under different slip conditions when M=0 and Λ=0.

Thus, Figure 2 presents a comparative illustration of the base flows corresponding to the three aforementioned slip scenarios of Newtonian fluids.

### 2.3. Linear Stability Analysis

Since Squire’s theorem is applicable to the slip flow modeled in this study, the following analysis is restricted to two-dimensional disturbances. To investigate the hydrodynamic stability of the problem, the instantaneous velocity and stress are expressed as the sum of the base state and the perturbation quantities. A linear stability analysis is performed on small perturbations to the basic flow:

(19) $$u=u_{b}+\varepsilon \hat{u},v=\varepsilon \hat{v},p=P+\varepsilon \hat{p}.$$

where ε≪1. Substituting Equation (19) into Equation (6) and linearizing with respect to ϵ, the linearized Navier–Stokes equations are obtained:

(20) 1−Λ∇2∂u∂t+ub∂u∂x+v∂ub∂y=−∂p∂x+1Re∇2u−M2Reu,1−Λ∇2∂v∂t+ub∂v∂x=−∂p∂y+1Re∇2v−M2Rev,∂u∂x+∂v∂y=0.

where $\nabla^{2}=\frac{\partial^{2}}{\partial x^{2}}+\frac{\partial^{2}}{\partial y^{2}}$. For simplicity, the hat symbols on the perturbation variables have been omitted, and primes denote differentiation with respect to *y*. A stream function ψ is introduced such that:

(21) $$u=\frac{\partial \psi }{\partial y},v=-\frac{\partial \psi }{\partial x}.$$

Assuming a wave-like solution of the form:

(22) $$\psi \left(x,y,t\right)=\phi \left(y\right)e^{i\left(\alpha x-\alpha ct\right)}.$$

For the streamwise wave number α∈R and phase velocity c∈C. Take the partial derivatives of the first two equations in Equation (20) with respect to *x* and *y* respectively, then subtract the resulting equations to eliminate the pressure-related terms. Finally, substitute Equations (21) and (22) into the pressure-eliminated equation, and Equation (23) can be obtained. When Λ=0, the equation reduces to the well-known Orr–Sommerfeld equation.

(23) ub−c1−ΛD2−α2D2−α2ϕ−D2ubϕ=1iαReD2−α22ϕ−M2iαReD2−α2ϕ.

The eigenvalue problem, Equation (23), is then solved subject to an equivalent set of boundary conditions:

(24) $$\phi =0,D\phi -\lambda_{1}D^{2}\phi =0\;\;\mathrm{on}\;\;y=-1.$$

(25) $$\phi =0,D\phi +\lambda_{2}D^{2}\phi =0\;\;\;\;\mathrm{on}\;\;y=1.$$

Herein, $u_{b}$ denotes the base flow, and $D^{2}$ represents the second derivative with respect to y. Consequently, in the temporal stability analysis with a real wave number α, the complex phase velocity $c=c_{r}+ic_{i}$ serves as the eigenvalue of the problem. Correspondingly, the flow exhibits linear stability when $c_{i}<0$ and linear instability when $c_{i}>0$.

The neutral curve constitutes a fundamental analytical tool in fluid stability studies, rooted in linear stability theory. This approach involves solving the eigenvalue problem of the Orr–Sommerfeld equation to determine the relationship between perturbation growth rates and flow parameters. In the (Re,α) plane, the neutral curve defined by the critical condition of zero growth rate ($c_{i}=0$) serves as a demarcation boundary separating stable ($c_{i}<0$, decaying perturbations) from unstable ($c_{i}>0$, growing perturbations) regimes. Systematic analysis of the neutral curve’s morphology and position enables determination of: (i) critical flow instability thresholds, (ii) dominant instability modes and their physical mechanisms, and (iii) laminar–turbulent transition criteria. Furthermore, the curve’s evolution reveals how stability characteristics are influenced by flow parameters including wall slip conditions, porosity, and viscoelastic coefficients, providing crucial theoretical insights for flow control strategies.

Numerically, the generalized eigenvalue problem is solved using the Chebyshev spectral collocation method. The interval $\left[-1,1\right]$ is discretized into N + 1 collocation points, and the disturbance velocity is expanded in a Chebyshev polynomial series. Substituting into the modified Orr–Sommerfeld equation leads to the discrete matrix system GX=cHX, where: G=ubD2−α2I−D2ubI−1iαReD4−2α2D2+α4I+M2iαReD2−α2I; $H=\left(D^{2}-\alpha^{2}I\right)-\Lambda \left(D^{4}-2\alpha^{2}D^{2}+\alpha^{4}I\right)$. The eigenvalue problem is solved using the QZ algorithm in MATLAB R2024b.

### 2.4. Analogy to the Poiseuille–Couette Flow

The Couette flow model can be regarded as a variant of asymmetric slip. After conducting a stability analysis on the base flow Equation (15), we solve the Orr–Sommerfeld equation Equation (23) under the condition of no applied slip.

(26) ϕ=0,Dϕ=0ony=1.

(27) ϕ=0,Dϕ=0ony=−1.

Pure Couette flow is characterized by a zero pressure drive, $\frac{\partial p}{\partial x}=0$. Poiseuille–Couette flow is distinguished by a constant pressure, $\frac{\partial p}{\partial x}=constant$.

This leads to the base flow equation:

(28) $$u_{b}=\frac{U}{2\cosh\left(M\right)}\cosh\left(My\right)+\frac{\cosh\left(M\right)}{\cosh\left(M\right)-1}\left(1-\frac{\cosh\left(My\right)}{\cosh\left(M\right)}\right)-\frac{U}{2\sinh\left(M\right)}\sinh\left(My\right).$$

When M→0

(29) $$u_{b}\left(y\right)=1-y^{2}+\frac{U}{2}\left(1-y\right).$$

where *U* is the velocity of the channel wall (in this particular model, the upstream channel wall is stationary). By comparing Equation (29) with the asymmetric base flow Equation (15),

$$\frac{U}{2}\left(1-y\right)=\frac{2\lambda_{0}\left(1-y\right)}{2+\lambda_{0}}$$

The following relationship between the wall velocity *U* and the slip length $\lambda_{0}$ is derived:

(30) $$U=\frac{4\lambda_{0}}{2+\lambda_{0}}.$$

This approach reveals a certain connection between this specific problem and Poiseuille–Couette flow. Thus, the linear stability of Poiseuille–Couette flow is equivalent to analyzing the asymmetric slip flow Equation (15) with the application of no-slip boundary conditions to the Orr–Sommerfeld Equation (23).

### 2.5. Disturbance Energy Budget Equation

To elucidate the physical mechanisms governing stability enhancement, we employ the classical Reynolds–Orr energy analysis method [1,23]. We take the inner product of the linearized perturbation momentum equation (Equation (20)) with the perturbation velocity vector V=(u,v), where *u* and *v* denote the streamwise and wall-normal disturbance components, respectively. Then, we integrate over the interval [−1,1] in the *y*-direction. This yields the rate of change in the total disturbance kinetic energy, defined as $E=\frac{1}{2}\int_{-1}^{1}\left(u^{2}+v^{2}\right)dy$. The derived energy budget equation for the NSV fluid in a porous medium is:

(31) $$\frac{dE}{dt}=P+D_{visc}+D_{porous}+W_{Voigt}.$$

Here, the terms on the right-hand side correspond to distinct physical processes:

$$P\left(y\right)=-\mathrm{real}\left(u\bar{v}\right)\frac{du_{b}}{dy},\;P=\int_{-1}^{1}P\left(y\right)dy$$

This term represents the rate at which the perturbation extracts energy from the base flow shear via Reynolds stress. Here, real(·) denotes the real part of a complex quantity, and the overbar $\bar{\left(\cdot \right)}$ denotes the complex conjugate. A positive value (P>0) serves as the primary source of instability.

$$D_{visc}\left(y\right)=-\frac{1}{Re}\left({\left|\nabla V\right|}^{2}\right),\;D_{visc}=\int_{-1}^{1}D_{visc}\left(y\right)dy$$

This term accounts for the kinetic energy loss caused by Newtonian viscosity. Mathematically, ${\left|\nabla V\right|}^{2}={\left|\frac{\partial u}{\partial x}\right|}^{2}+{\left|\frac{\partial u}{\partial y}\right|}^{2}+{\left|\frac{\partial v}{\partial x}\right|}^{2}+{\left|\frac{\partial v}{\partial y}\right|}^{2}$ represents the total intensity of fluid deformation. It is strictly negative, indicating a stabilizing effect.

$$D_{porous}\left(y\right)=-\frac{M^{2}}{Re}\left(u^{2}+v^{2}\right),\;D_{porous}=\int_{-1}^{1}D_{porous}\left(y\right)dy$$

Unique to this study, this term quantifies the Darcy drag exerted by the porous matrix. It functions as an additional energy sink, uniformly suppressing disturbances across the channel.

$$W_{Voigt}\left(y\right)=\mathrm{real}\left(\Lambda V\cdot \partial_{t}\left(\nabla^{2}\bar{V}\right)\right),\;W_{Voigt}=\int_{-1}^{1}W_{Voigt}\left(y\right)dy$$

This term captures the contribution of the Voigt viscoelastic term.

In linear stability analysis, it acts as a regularization mechanism that smooths out high-frequency oscillations. Under the framework of linear stability theory, the disturbance growth rate $c_{i}$ is determined by the net energy budget. Instability occurs only when the production P exceeds the sum of all dissipation mechanisms.

### 2.6. Physical Parameter Selection

The key dimensionless parameters in this study are selected based on physical feasibility and experimental references:

Voigt parameter (Λ): Defined as $\Lambda =\frac{\tilde{\lambda }}{h^{2}}$ ($\tilde{\lambda }$ is the dimensional Kelvin–Voigt elastic parameter, *h* is the channel half-width). For typical viscoelastic fluids (e.g., PEO/PAM aqueous solutions) with relaxation time $\tilde{\lambda }=1\times 10^{-6}$– $1\times 10^{-3}$ s [27], and microchannel half-width $h=1\times 10^{-4}$–$1\times 10^{-3}$ m, Λ ranges from $10^{-8}$ to $10^{-5}$.

Permeability parameter (*M*): Expressed as $M=\frac{h\phi }{k}$ (ϕ is porosity, *k* is permeability). Covering scenarios from pure fluids (M=0, k→∞) to low-permeability porous media (M=3), it corresponds to porosity ϕ=0.3–0.5 and permeability $k\approx 10^{-14}$–$10^{-10}\;m^{2}$, typical of soil, sandstone, and shale [28].

Slip length ($\lambda_{1},\lambda_{2}$): Dimensionless slip length is the ratio of physical slip length to h. Physical slip lengths of 1–10 μm (consistent with experimental measurements [9,10]) correspond to λ = 0–0.05 for $h=1\times 10^{-4}$ m.

## 3. Discussion of Result

### 3.1. Numerical Convergence and Validation

Next, a convergence test via eigenvalue solution is performed for the Navier–Stokes–Voigt fluid under the conditions of $\Lambda =10^{-6}$, M=1, $\lambda_{1}=0.01$, and $\lambda_{2}=0.05$. For the selected parameters (Re=11,000, α=1), the convergence test for the least stable eigenmode ($c_{i}>0$) is presented with an increasing number of collocation points. Table 1 first conducts the convergence test for the least stable eigenmode of Newtonian fluid under no-slip boundary conditions. Subsequently, the convergence test is also carried out for the case of $\Lambda =10^{-6}$, M=1, and $\lambda_{1}=\lambda_{2}=0.05$. This table confirms that when the number of collocation points N=150, the numerical scheme achieves good convergence with at least six decimal places. These convergence results lay a benchmark for the subsequent comprehensive analysis. Furthermore, in comparison with the data of Newtonian fluid under no-slip boundary conditions (Re=6000, α=1) reported by Ceccacci et al. [29], the least stable eigenmode obtained via the numerical method in this study is 0.259816+0.000323i, which is in perfect agreement with their results up to six decimal places. Compared with the least stable eigenmode of 0.217242−0.156831i reported by Arjun et al. [30] for Navier–Stokes–Voigt fluid with $\Lambda =1\times 10^{-6}$, M=1, $\lambda_{1}=\lambda_{2}=0.03$ (Re =10,000, α=1), our result also shows complete consistency at the six-decimal-place level.

### 3.2. Compare with Poiseuille–Couette Flow

**Figure 3:**
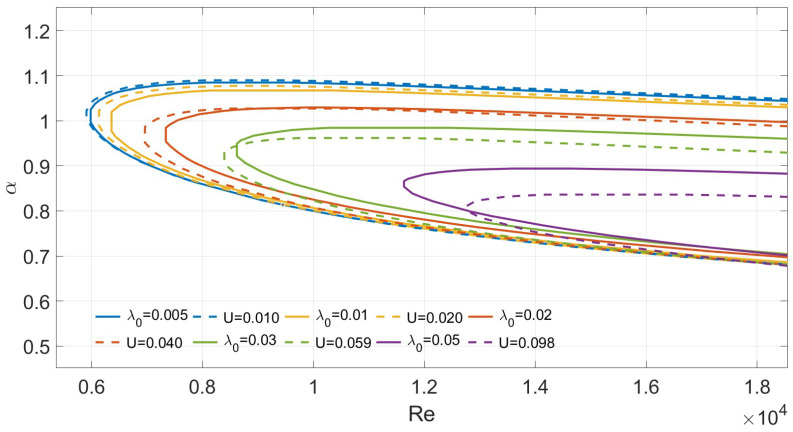
Neutral curves for comparision between Couette and slip models when M=Λ=0.

**Figure 4:**
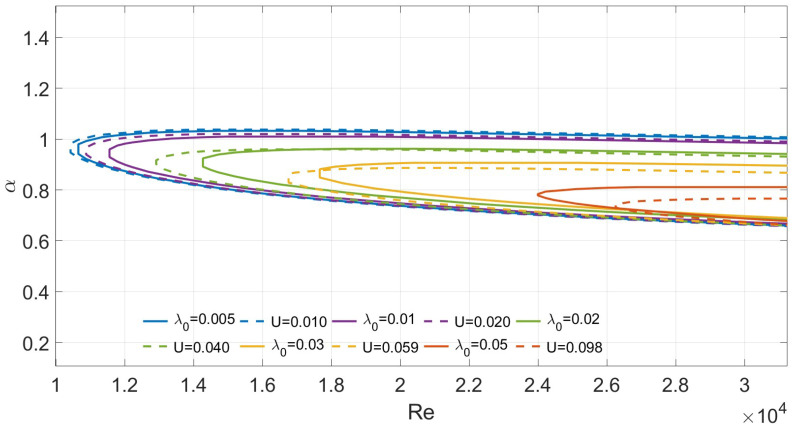
Neutral curves for comparision between Couette and slip models when M=1, $\Lambda =1\times 10^{-7}$.

When M=0 and Λ=0, the fluid behaves as a Newtonian fluid. Figure 3 illustrates the Poiseuille–Couette flow with one-sided slip under the conditions of M=0 and Λ=0. Values of $\lambda_{0}$ are taken as 0.005,0.01,0.02,0.03,0.05 and the corresponding comparisons with the Couette flow satisfying Equation (30) are plotted. As $\lambda_{0}$ decreases, the two curves tend to coincide. With increasing $\lambda_{0}$, although *U* still varies proportionally, a certain discrepancy in the critical Reynolds number emerges. Figure 4 compares the two flow models for a Navier–Stokes–Voigt fluid with M=1 and $\Lambda =1\times 10^{-7}$, under the condition that the relationship between the slip length $\lambda_{0}$ and the Couette plate velocity *U*, as previously described, remains approximately valid. The discrepancy between the two models again increases with increasing $\lambda_{0}$. It is found that this approach is only suitable for small slip-length approximations. By performing a Taylor expansion of Equation (30), it is observed that both models exhibit identical linearized behavior to first-order approximation. However, when $\lambda_{0}>0.01$, terms of order $\lambda_{0}^{2}$ and higher become non-negligible. For large $\lambda_{0}$, the discretization error of Chebyshev collocation points near the boundary is amplified, and the condition number of the generalized eigenvalue problem deteriorates. Therefore, the optimal range of agreement is found to be $\lambda_{0}\in \left[0,0.01\right]$. This study is also relevant to stability analysis in the context of superhydrophobic surfaces: when the material slip length is small, the stability of such flows may be approximated by that of Couette flow.

### 3.3. Eigenspectrum

**Figure 5:**
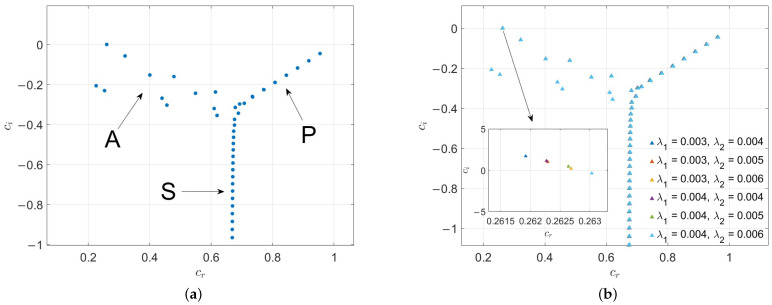
Comparison of the eigenvalue spectra for Newtonian fluids at Re=6000 and α=1. (**a**) No-slip boundary conditions. (**b**) Varying slip lengths $\lambda_{1}$ and $\lambda_{2}$.

Figure 5 compares the eigenvalue spectra for Newtonian fluids (M=Λ=0) at Re=6000 and α=1 under both no-slip and slip conditions. As shown in Figure 5a, when no-slip boundaries are examined, the classic Newtonian spectrum exhibits a characteristic Y-shaped structure. This structure consists of three distinct branches—A, P, and S—collectively referred to as the APS branches [31]. In Figure 5b, we introduce slip boundary conditions. The results demonstrate that introducing wall slip causes the A-branch to shift downward into the stable half-plane ($c_{i}<0$), which aligns with the stabilizing effect reported in previous literature. The APS branches are three types of modes in the spectrum of Newtonian fluids, classified based on the distribution of perturbation energy, the trend of phase velocity, and stability behavior. The A branch corresponds to wall modes, which are perturbation modes dominated by the shear effect of the flow wall. The phase velocity *c* tends to 0, so the perturbation propagates extremely slowly, almost “attached” near the wall. They are usually damped modes $c_{i}<0$, but under specific flow conditions, they may transform into growing modes, triggering flow instability near the wall. Changing the magnitude of some influencing factors can easily make them unstable or restore their stability. The P branch corresponds to central modes, which are perturbation modes dominated by the core region of the flow, with energy mainly concentrated in the central region where the flow velocity is relatively high. The phase velocity tends to the maximum velocity of the base flow, so the perturbation propagates at a speed close to the mainstream velocity. They may exhibit as growing modes $c_{i}>0$ or damped modes, and are key modes governing the global stability of the flow. The S branch corresponds to damped modes, which is the third family of eigenvalues derived by Schensted [32]. This family has an infinite range, with two asymptotic expressions for its eigenvalues. One is the formula proposed by Schensted: ci=−n2π2αRe (where $c_{r}$ is unspecified), and the other is the formula put forward by Grosch and Salwen [33] in their extensive study of the spectrum of plane Poiseuille flow: −4n−1216αRe. According to Grosch and Salwen, the asymptotic value of $c_{r}$ is $\frac{2}{3}$ of the average velocity of the mean flow in the channel.

**Figure 6:**
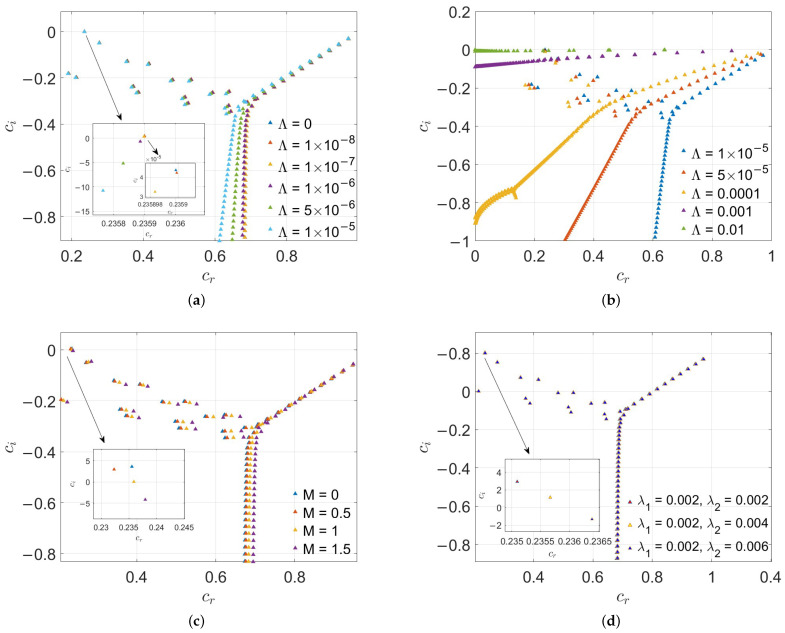
Variations in the eigenvalue spectrum with respect to different control parameters. (**a**) Varying Voigt parameter Λ from 0 to $1\times 10^{-5}$ with $\lambda_{1}=0.004,\lambda_{2}=0.003,M=1,Re$ = 11,000, α=1. (**b**) Varying Voigt parameter Λ from $1\times 10^{-5}$ to 0.01 with $\lambda_{1}=0.004,\lambda_{2}=0.003,M=1,Re$ = 11,000, α=1. (**c**) Varying porous parameter *M* with $\lambda_{1}=0.004,\lambda_{2}=0.003,\Lambda =1\times 10^{-7},Re$ = 11,000, α=1. (**d**) Varying the upper wall slip length $\lambda_{2}$ from 0.002 to 0.006 with a fixed lower wall slip $\lambda_{1}=0.002,\Lambda =1\times 10^{-7},M=1,Re$ = 11,000, α=1.

Figure 6a presents a configuration with an upper-wall slip length of $\lambda_{2}=0.003$, a lower-wall slip length of $\lambda_{1}=0.004$, Re = 11,000, and α=1. As the viscoelastic parameter Λ increases from 0 to $1\times 10^{-5}$, the locally magnified view reveals that the most unstable mode crosses the $c_{i}$-axis, indicating enhanced flow stability with increasing Λ. However, it is observed that when Λ increases to $5\times 10^{-6}$, the S-branch begins to shift leftward. A further increase in Λ to $1\times 10^{-5}$ results in a more pronounced leftward translation of the S-branch. Examination of the local detailed view shows that the most unstable mode now lies below zero. Moreover, even if slip and porosity effects are reduced, the flow remains stable under these conditions. This behavior is consistent with the findings reported by Shankar [23] et al.

Figure 6b explores the eigenvalue spectrum of NSV fluids with larger Voigt parameters (up to Λ=0.01). In some non-Newtonian models, increasing viscoelastic parameters triggers severe instability. However, the results for the NSV model are different. As Λ increases, the traditional Y-shaped eigenvalue branches collapse toward the real axis. These branches remain strictly within the stable half-plane ($c_{i}\leq 0$). This confirms that the Voigt term acts as a consistent stabilizing factor.

Figure 6c investigates the effect of the permeability coefficient of porous media on the asymmetric slip flow of viscoelastic fluids. With $\Lambda =1\times 10^{-7}$, $\lambda_{1}=0.004,\lambda_{2}=0.003$, Re = 11,000, and α=1 kept constant, as *M* increases from 0 to 1.5, it is noted that the unstable mode smoothly moves below $c_{i}=0$, as shown in the local enlarged view. This confirms the stabilizing effect of increasing the porous medium parameter *M* on the flow.

The influence of slip length on the flow of Navier–Stokes–Voigt fluids is illustrated in Figure 6d. With M=1 and $\Lambda =1\times 10^{-7}$ fixed, the lower plate has a slip length of $\lambda_{1}=0.002$, while the upper plate slip length $\lambda_{2}$ is varied from 0.002 to 0.006. The results clearly demonstrate that increasing the slip length plays a significant role in enhancing the stability of this viscoelastic fluid. In summary, the unstable modes in all the aforementioned cases belong to the *A*-branch.

Finally, a direct comparison between Figure 5b and Figure 6 robustly validates our core conclusions. In the purely Newtonian regime without a porous medium (M=0,Λ=0, as shown in Figure 5b), the specific asymmetric slip configuration ($\lambda_{1}=0.004,\lambda_{2}=0.003$) yields a maximum eigenvalue $c_{i}>0$, indicating an unstable flow. However, as demonstrated in Figure 6a,c, introducing and increasing either the viscoelastic parameter Λ or the porous medium parameter *M* under the identical asymmetric slip condition systematically shifts the most unstable mode back into the stable half-plane ($c_{i}\leq 0$). This comparison firmly establishes the powerful stabilizing roles of both the Voigt regularization and the Darcy dissipation in further enhancing the overall stability of the fluid system.

### 3.4. Neutral Stability Curves

**Figure 7:**
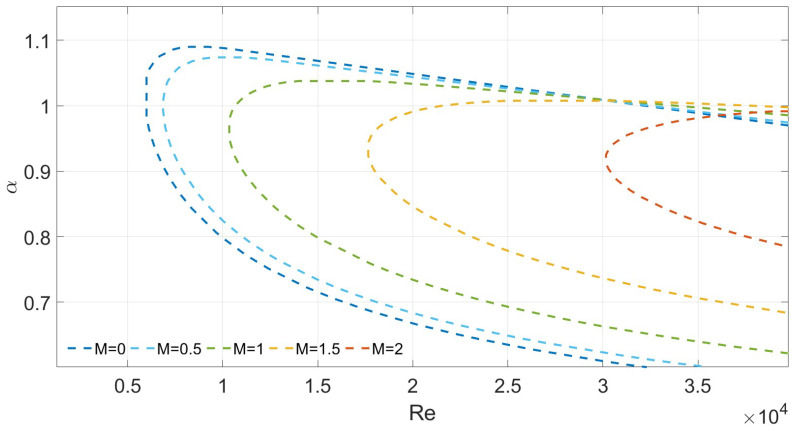
Neutral curves for varying *M* for $\lambda_{1}=\lambda_{2}=0.002$ and Λ=0.

To verify the reliability of the numerical scheme, we compare the predicted critical wavenumber ($\alpha_{c}$) and critical Reynolds number ($Re_{c}$) for Poiseuille flow in a porous medium under symmetric slip conditions with results from the literature. For validation, parameters Λ=0 and $\lambda_{1}=\lambda_{2}=0.002$ are used. As shown in Figure 7, as the porous medium parameter *M* increases from 0 to 2, the computed values of $\alpha_{c}$ and $Re_{c}$ (e.g., $Re_{c}\approx$ 10,158 at M=1) agree well with those reported by Straughan and Harfash. This confirms the accuracy and reliability of the numerical implementation.

**Figure 8:**
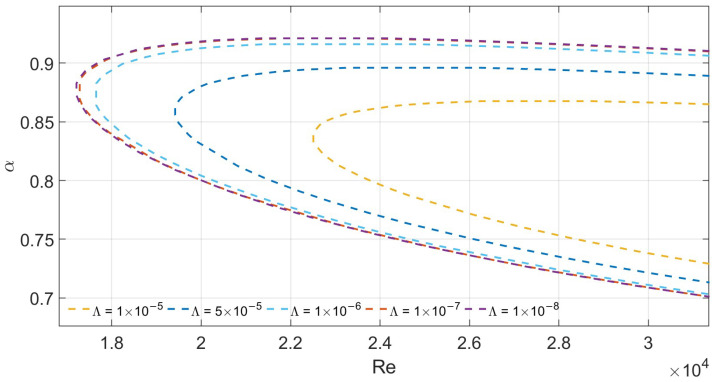
Neutral curves for varying Λ for M=1 and $\lambda_{1}=0.01,\lambda_{2}=0.02$.

We next investigate the influence of various parameters on flow stability when the top and bottom wall slip lengths are asymmetric. Figure 8 shows the variation in the critical Reynolds number with Λ for M=1, $\lambda_{1}=0.01$, and $\lambda_{2}=0.02$. The results indicate that $Re_{c}$ increases with Λ.

**Figure 9:**
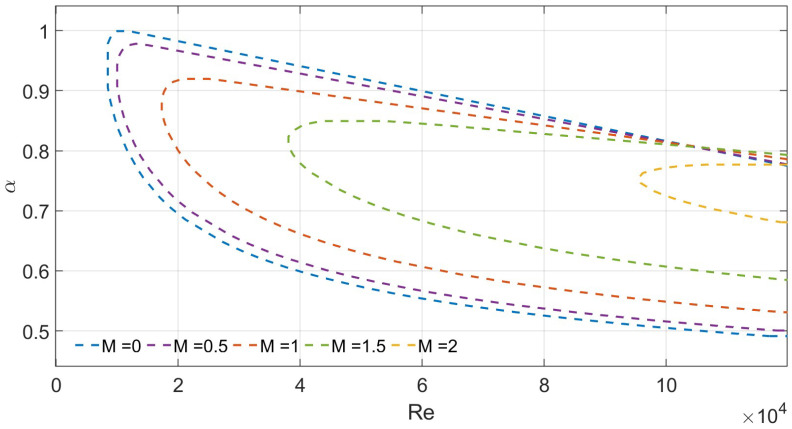
Neutral curves for varying *M* for $\lambda_{1}=0.01$, $\lambda_{2}=0.02$ and $\Lambda =1\times 10^{-6}$.

Figure 9 presents the variation in $Re_{c}$ and $\alpha_{c}$ with *M* for $\Lambda =1\times 10^{-6}$, $\lambda_{1}=0.01$, and $\lambda_{2}=0.02$. When M=2, the critical Reynolds number exceeds $1\times 10^{5}$, which is two orders of magnitude higher than the reference case with M=0, Λ=0, and no-slip boundaries ($Re_{c}=5772.2$). This indicates that the combined effect of wall slip and porous media significantly raises the instability threshold of NSV fluid flow. Numerical results show that increasing *M* (which inherently couples porosity and permeability) leads to a significant rise in the critical Reynolds number $Re_{c}$. This indicates that Darcy dissipation in porous media is a powerful mechanism for suppressing disturbance growth. However, it should be noted that both *M* and Λ are dimensionless parameters. Their relative importance depends heavily on specific physical applications and the chosen parameter ranges. Therefore, this comparison only highlights the qualitative differences between the two mechanisms rather than ranking their importance.

**Figure 10:**
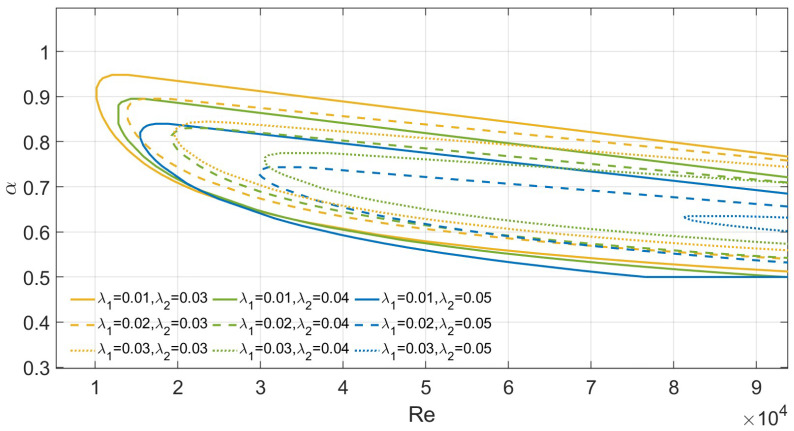
Neutral curves for varying λ for $\Lambda =1\times 10^{-7}$ and M=0.1.

Finally, Figure 10 presents the neutral stability curves for $\Lambda =1\times 10^{-7}$, M=0.1, and unequal slip lengths ($\lambda_{1}\neq \lambda_{2}$). The numerical results indicate that increasing the slip length on either boundary fundamentally stabilizes the flow. For example, when $\lambda_{1}=0.03$ and $\lambda_{2}=0.05$, $Re_{c}$ = 81,024.5. Additionally, we found that for a fixed total slip length, a more symmetric slip distribution results in higher flow stability. For instance, the symmetric case with $\lambda_{1}=\lambda_{2}=0.03$ yields a higher critical Reynolds number ($Re_{c}$) than the asymmetric case with $\lambda_{1}=0.01$ and $\lambda_{2}=0.05$.

### 3.5. Energy Budget Analysis

To elucidate the physical mechanisms governing the competition between destabilizing and stabilizing forces, we performed a global energy budget analysis.

**Figure 11:**
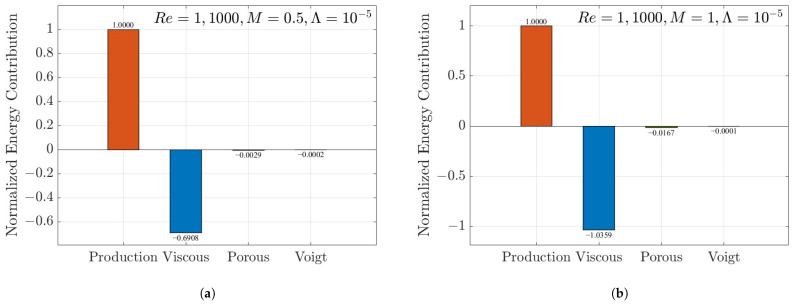
Comparison of the global energy budgets for the critical mode under asymmetric slip conditions ($\lambda_{1}=0.003,\lambda_{2}=0.001$). (**a**) The flow is unstable at M=0.5. (**b**) The flow becomes stable at M=1.

Figure 11a,b present the normalized contributions of the Reynolds stress production (P), viscous dissipation ($D_{visc}$), porous damping ($D_{porous}$), and Voigt regularization work ($W_{Voigt}$) to the time rate of change in the disturbance kinetic energy (dE/dt). As shown in Figure 11a, the flow exhibits a distinct energy imbalance at a lower porous parameter of M=0.5. The Reynolds stress production (orange bar, normalized to 1.0) extracts energy from the asymmetric mean shear. Its magnitude exceeds the sum of all dissipative mechanisms. Specifically, the viscous dissipation (blue bar, −0.6908), combined with the minor porous damping and Voigt terms, is insufficient to counteract the energy production. This results in a net energy surplus (dE/dt>0). This result physically confirms the occurrence of linear instability driven by the asymmetric boundary conditions. In contrast, increasing the porous parameter to M=1 significantly alters the energy balance, as illustrated in Figure 11b. The stronger interaction between the fluid and the porous matrix modifies the perturbation mode shape. This leads to a substantial enhancement in viscous dissipation (magnitude increases to 1.0359). Consequently, the total dissipation overcomes the energy production, resulting in a net energy decay (dE/dt<0). This demonstrates that the porous medium acts as a critical stabilizing factor. When *M* is sufficiently large, it suppresses the instability induced by the slip. It is noteworthy that the Voigt term consistently makes a negative contribution in both stable and unstable regimes, despite its small magnitude (order of $10^{-4}$). This confirms that the Voigt term functions primarily as an auxiliary energy sink (dissipative regularization) rather than an elastic energy source, regardless of the flow’s stability status.

To uncover the physical origin of the destabilizing effect observed in the neutral curves, we examine the spatial distribution of the energy production term, P(y). This term represents the rate of energy transfer from the mean flow to the disturbance.

**Figure 12:**
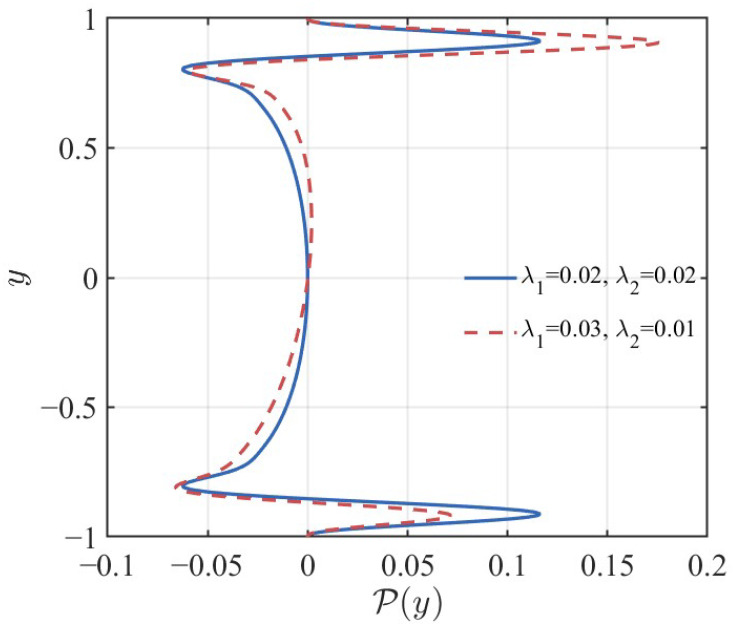
Comparison of the spatial distribution of the energy production term P(y) between symmetric ($\lambda_{1}=\lambda_{2}=0.02$) and asymmetric ($\lambda_{1}=0.03,\lambda_{2}=0.01$) slip conditions at Re = 11,000 and M=1.

Figure 12 compares the production profiles between the symmetric case ($\lambda_{1}=\lambda_{2}=0.02$) and the asymmetric case ($\lambda_{1}=0.03,\lambda_{2}=0.01$) at Re = 11,000 and M=1. In the symmetric configuration (blue solid line), the energy production exhibits a balanced, bi-modal distribution. Two equal peaks are located near the critical layers at both walls. This symmetry indicates that the destabilizing potential is evenly distributed across the channel. However, the introduction of slip asymmetry (red dashed line) drastically alters this balance. The profile becomes highly skewed. It is characterized by a dominant peak near the upper wall (y≈0.9), where the slip length is smaller ($\lambda_{2}=0.01$). Fluid tends to flow more easily over the wall with higher slip ($\lambda_{1}=0.03$). This causes the maximum velocity of the base flow to shift towards the lower wall. Consequently, to satisfy mass conservation and boundary conditions, the mean velocity gradient (shear rate, dU/dy) steepens significantly near the opposite wall (the upper wall). As evidenced in Figure 12, the peak production in the asymmetric case significantly exceeds the maximum value observed in the symmetric case. This localized surge acts as a powerful source of instability. It injects energy into the disturbance field at a rate that the global viscous and Voigt dissipation mechanisms cannot effectively counteract. This triggers the onset of instability at lower Reynolds numbers, as predicted by the neutral curves.

**Figure 13:**
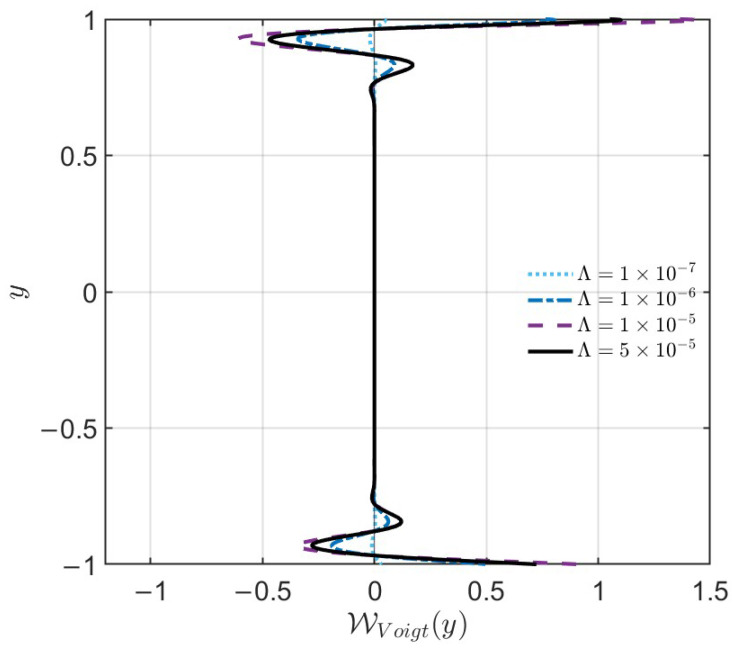
Spatial distribution of the normalized Voigt regularization work density $W_{Voigt}\left(y\right)$ for varying Voigt parameters Λ ($10^{-7},10^{-6},10^{-5},5\times 10^{-5}$). The fixed parameters are Re = 11,000, M=1, and asymmetric slip conditions $\lambda_{1}=0.03,\lambda_{2}=0.01$.

As shown in the Figure 13, the Voigt regularization work $W_{Voigt}\left(y\right)$ is not uniformly distributed across the channel; instead, it is highly concentrated near the walls, targeting the regions with steep velocity gradients. Consistent with the mathematical definition of the Voigt term, as the parameter Λ increases, the amplitude of this local regularization work grows significantly, resulting in sharper and more pronounced dissipative peaks near the boundaries (e.g., for $\Lambda =5\times 10^{-5}$). As Λ increases, the Voigt term acts as a targeted energy sink, inherently magnifying the localized dissipation precisely at the near-wall regions where the asymmetric slip induces the maximum shear and Reynolds stress production. Ultimately, this enhanced, localized energy sink becomes strong enough to fully suppress the instability driven by the asymmetric shear, stabilizing the global flow. Crucially, as shown in the global energy budget analysis (Figure 11), the net integration of this profile yields a negative value. This confirms that despite local fluctuations, the global effect of the localized Voigt term is to dissipate kinetic energy. It effectively suppresses wall-bounded disturbances, thereby stabilizing the flow.

## 4. Conclusions

This study systematically investigates the linear stability of Navier–Stokes–Voigt (NSV) fluid flow in porous media under general asymmetric slip boundary conditions. By extending the classical analysis of symmetric slip boundaries to asymmetric ones, and supported by both numerical evidence and energy budget analysis, the core conclusions are summarized as follows:

First, key parameters demonstrate a consistent stabilizing effect on the flow. Increasing the Voigt parameter Λ or the porous medium parameter *M* significantly raises the critical Reynolds number $Re_{c}$. For a high-permeability medium (M=2), the stability threshold exceeds $Re_{c}=1\times 10^{5}$. This is two orders of magnitude higher than the critical value for Newtonian fluids under no-slip conditions ($Re_{c}=5772.2$). Notably, the flow becomes unconditionally stable when $\Lambda \geq 1\times 10^{-5}$. Furthermore, comparing the stability characteristics of the NSV model with recent non-Newtonian models provides new insights. For instance, Sun [34] demonstrated a specific phenomenon in certain non-Newtonian fluids, such as those governed by second-order nonlinear spatial viscosity terms. In these fluids, increasing the non-Newtonian parameter beyond a critical threshold causes entire eigenvalue branches to enter the unstable region. This triggers extreme flow complexities and turbulence. In stark contrast, our eigenspectrum (Figure 6b) and energy analyses (Figure 11) confirm that the NSV fluid behaves in a completely opposite manner. Applying an extremely large Λ does not induce turbulence. Instead, it unconditionally maintains flow stability. Energy analysis reveals the underlying physical mechanism. The Voigt term consistently acts as a dissipative energy sink (a regularization term) rather than an elastic energy source. Working together with the Darcy drag from the porous medium, it effectively suppresses disturbances near the wall.

Second, an important finding of this study is the impact of wall slip on stability. Introducing or increasing wall slip clearly stabilizes the overall flow. However, the system is relatively sensitive to how the slip is distributed. It must be emphasized that independently increasing the slip length on either wall enhances fluid stability. For example, in Figure 10, the neutral curves shift to the right when $\lambda_{1}$ is fixed and $\lambda_{2}$ is increased. However, our research also offers an optimal solution for a practical engineering problem: how to maximize flow stability when applying a fixed amount of superhydrophobic coating to a microchannel. A fixed amount of coating material represents a constant total slip in our model (i.e., $\lambda_{1}+\lambda_{2}=\mathrm{constant}$). When evaluating the slip distribution under this premise, symmetric slip provides the optimal stability margin. For instance, as also shown in Figure 10, for a total slip length of $\lambda_{\mathrm{total}}=0.06$, the critical Reynolds number $Re_{c}$ for the symmetric distribution ($\lambda_{1}=0.03,\;\lambda_{2}=0.03$) is significantly higher than that for the asymmetric counterpart ($\lambda_{1}=0.01,\;\lambda_{2}=0.05$). Energy analysis clarifies the physical root of this phenomenon. Asymmetry distorts the base flow shear. This creates a localized surge in Reynolds stress energy production. Consequently, any deviation from symmetry prevents the system from fully maximizing the stabilizing potential of a given slip budget. Thus, instability is triggered earlier than in a symmetric configuration with the exact same total slip. Furthermore, as the degree of asymmetry increases, the trend of stability reduction gradually flattens. This indicates a saturation of this destabilizing effect.

Furthermore, as the degree of asymmetry increases, the trend of stability reduction gradually flattens, indicating a saturation of the destabilizing effect. Third, from an engineering perspective, these findings establish theoretical bounds for manufacturing tolerances in superhydrophobic channels. While uniform coating is ideal, our results indicate that performance degradation caused by unavoidable manufacturing asymmetry can be compensated. This is achieved by slightly increasing the porous medium parameter (*M*) or the Voigt regularization parameter (Λ). This offers a robust design strategy for microfluidic chips and hemodialyzers, ensuring flow stability even when coating uniformity is imperfect. Fourth, we clarified the applicability boundaries of the simplified analogy between asymmetric slip flow and Poiseuille–Couette flow. This analogy is valid only for small slip lengths ($\lambda_{0}\leq 0.01$, first-order approximation). For larger slip lengths ($\lambda_{0}>0.01$), higher-order terms become non-negligible, making the analogy suitable only for qualitative verification rather than quantitative prediction. Additionally, the numerical scheme demonstrates high robustness, achieving high accuracy (error ≤5%) with N=150 collocation points. However, for the large slip regime (λ>0.02), we recommend using N=200 to address numerical sensitivity.

Finally, regarding contributions and future outlook, this study advances existing research by moving beyond the idealized symmetric assumption of Shankar and Shivakumara [23]. It also extends the analytical scope of Baranovskii [18,19] by incorporating stability criteria in porous media. Future research should extend the model to full second-grade fluid or turbulent flow systems. This will allow for exploring the nonlinear coupling between slip asymmetry and viscoelasticity, which should be validated through experimental measurements.

## Figures and Tables

**Table 1:** Presentation of the convergent values of the unstable modes for the plane Poiseuille flow for (Re=11,000) and (α=1) with increase in collocation points.

N	$\Lambda =M=\lambda_{1}=\lambda_{2}=0$	$\Lambda =1\times 10^{-6},M=1,\lambda_{1}=0.01,\lambda_{2}=0.05$	$\Lambda =1\times 10^{-6},M=1,\lambda_{1}=\lambda_{2}=0.05$
50	0.233537+0.004010i	0.266767−0.008964i	0.297008−0.014072i
100	0.233538+0.004011i	0.266775−0.008962i	0.297038−0.014096i
150	0.233538+0.004011i	0.266775−0.008962i	0.297038−0.014096i

## Data Availability

The data presented in this study are available on request from the corresponding author.
